# Supplementary material for: Family planning demand generation in Rwanda: Government efforts at the national and community level impact interpersonal communication and family norms
Source: PLoS One. 2022 Apr 7;17(4):e0266520. doi: 10.1371/journal.pone.0266520 (PMC8989356; doi:10.1371/journal.pone.0266520)
Supplement: Attachment — Submitted filename: PONE-D-21-23342_review.docx [file pone.0266520.s001.docx]

This is an important topic. The authors conducted a qualitative study to examine the Rwandan government’s efforts to increase the demand of family planning methods and the resulting effect on community-level and individual level perceptions on contraceptives. The paper does make some good contributions but for the lessons from Rwanda to be understood clearly and applied elsewhere, there are some concerns that should be fixed prior to possible publication. It will also be nice if authors can clearly articulate what novel and/or surprising findings they are contributing.

Line 66 please specify year

Line 77 in their conclusion, the authors state that they used the diffusions of innovations theory as a framework of discussing the present work. However, as a reviewer, it was not clear to me from the introduction what this theory is and I was therefore not able to assess properly how it ties into the important topic discussed here.

Line 85 Authors should qualify what kind of messages they are referring to

Line 92 please clarify: past few months relative to what?

Line 95 I understand that the paper is focused on family planning but I think it would be good for the readers to be clear that Umuganda is not a day for family planning as the sentence currently reads. It is a community day that where a range of information is shared *including* family planning.

Line 97 redundant information on the 5 years. Also authors should clarify whether the contraceptive prevalence is referring to modern contraceptives. Please be sure to specify throughout paper including in abstract. Also after all of the statistics provided, the reader is left wondering what exactly these challenges that have led to the stagnating contraceptive prevalence are. A sentence or 2 providing additional context would suffice. Also something to consider, Rwanda had a new DHS survey (fieldwork during November 2019 - July 2020). The statistics provided may have changed and the fieldwork timeframe of new Rwanda DHS is closer to when authors did their interviews.

Line 104 increase prevalence instead of advance? Also this paragraph needs to be anchored a bit more into the Rwanda context.

Line 121 unclear who the interviewers were, how they were recruited and their training

Line 127 is thematic saturation a method or an endpoint? The authors should revise this sentence for clarity.

Line 175 overwhelmingly positively?

Line 246 revise sentence as the quote is unclear as currently written.

Line 258 the authors should be consistent in the tense they use in their writing. Please revise. Same as line 287.

Line 398 thinking about how these findings may be used elsewhere, how does Rwanda rank in terms of radio use relative to other countries in the region?

Lines 423-429 information is redundant with previous paragraph. It is not clear what this paragraph is specifically adding.

Lines 430 – 436 the authors should engage with the literature and comment whether this has been observed elsewhere

Line 472 why cite only CHWs and leave out nurses here?

Lines 474- 476 Authors should comment somewhere whether findings are as applicable in rural vs urban settings. ie are Musanze and Nyamasheke more rural, urban? It was interesting in the introduction that the authors provided information that contraceptive uptake also increased by 36% among rural women but it would be good to situate the study sites as well.
